# Elucidating the interplay between intracellular manganese–iron-ratio and radiotolerance across all domains of life

**DOI:** 10.1093/femsmc/xtag029

**Published:** 2026-05-28

**Authors:** Kristina Beblo-Vranesevic, Hector Palomeque-Dominguez, Tommaso Zaccaria, Robert Reichelt, Dina Grohmann, Jessica A Stammeier, Petra Rettberg

**Affiliations:** German Aerospace Center (DLR), Institute of Aerospace Medicine, 51147 Cologne, Germany; German Aerospace Center (DLR), Institute of Aerospace Medicine, 51147 Cologne, Germany; German Aerospace Center (DLR), Institute of Aerospace Medicine, 51147 Cologne, Germany; Department of Internal Medicine, Radboud University Medical Center, 6525 GA Nijmegen, The Netherland; Faculty for Biology and Preclinical Medicine, Institute of Microbiology and Archaea Centre, University of Regensburg, 93040 Regensburg, Germany; Faculty for Biology and Preclinical Medicine, Institute of Microbiology and Archaea Centre, University of Regensburg, 93040 Regensburg, Germany; GFZ Helmholtz Centre for Geosciences, 14467 Potsdam, Germany; German Aerospace Center (DLR), Institute of Aerospace Medicine, 51147 Cologne, Germany

**Keywords:** Mn/Fe-ratio, ionizing radiation, reactive oxygen species (ROS), extremophiles, radiation resistance

## Abstract

Exposure to natural or artificial ionizing radiation induces direct damage to intracellular macromolecules and promotes the formation of reactive oxygen species, which can further amplify cellular injury. Although many organisms tolerate high radiation doses, the basis of this resilience is not fully understood. A proposed determinant is the intracellular manganese-to-iron ratio (Mn/Fe-ratio), thought to reduce oxidative stress. To evaluate its relevance, Mn and Fe levels were measured in 19 archaeal, bacterial, and two eukaryotic yeast species and compared these values with their survivability after ionizing radiation exposure. There was no consistent relationship between Mn/Fe-ratios and radiation tolerance across this broad phylogenetic range. Integrating our results with prior studies suggests that while elevated Mn/Fe-ratios may contribute to exceptional resistance in certain specialized microorganisms, the ratio is not a reliable, universal predictor of survivability. These findings highlight the multifactorial nature of radiation tolerance and indicate that other processes (e.g. DNA repair mechanisms, antioxidant defenses, and metabolic adaptations) likely play central roles. By clarifying the limitations of Mn/Fe-ratio as a generalizable marker, this study provides a more nuanced understanding of the biochemical determinants of radiation resistance and informs future research into mechanisms of cellular resilience under extreme stress.

## Teaser

Microbes can sometimes survive radiation by maintaining a certain balance of manganese and iron, but our study shows that many other strategies also help cells withstand such extreme stress.

## Introduction

Astrobiology is the scientific study of life in the universe—its origins, evolution, and potential distribution—and examines how microorganisms use diverse cellular adaptation mechanisms to survive in extreme environments both on Earth and potentially beyond (Horneck et al. 2010). Extremophilic (micro-) organisms have entered an evolutionary path that allows them to thrive under extreme conditions even to the extent that they strictly depend on the extreme parameters in their natural habitat (Rothschild and Mancinelli 2001, Rampelotto 2013). Among these extremophiles, Archaea are thought to be among the earliest cellular life-forms on Earth, representing a very ancient lineage well adapted to primordial extreme environments (Williams et al. 2013, Caetano-Anollés et al. 2014). These cellular adaptation mechanisms, which enable microorganisms to withstand multiple types of environmental stress, are of particular relevance to astrobiology because they help define the limits of life and the conditions under which life might persist beyond Earth. These extreme conditions, both natural and artificial, include a combination of extreme chemical conditions, e.g. low or high pH, high salinity; or physical conditions, such as high pressure, cold or hot temperatures, low water activity, or high radiation levels. Low doses of radiation, here ionizing radiation, can be detected in certain areas with the occurrence of special mineralogical compounds. For example, at beach sands in Southeastern Brazil, natural radioactivity has been measured in the range of nano-Gray [45 –100 nGy/h; (Licínio et al. 2021)]. In contrast, high doses (in the kilo-Gray range) of ionizing radiation occur on Earth only in artificial environments. For example, doses exceeding 20 kGy are used in industrial applications, such as sterilization of food (Thomas 1988, McKeen 2012, Josephson and Peterson 2018).

The key question is, what happens in the microbial cell when it is exposed to low, non-lethal doses of ionizing radiation? Exposure to ionizing radiation causes direct effects on cellular components, in addition to indirect effects due to radiolysis of (intracellular) water molecules. Direct effects occur when the energy of ionizing radiation is absorbed by DNA, leading to excitation or direct ionization of the molecule with the possible consequence of DNA single- or double-strand breaks, base damage, or base loss, or cross-links within DNA or with neighboring proteins (Roobol et al. 2020). However, damages can also occur if other macromolecules (proteins, lipids) are hit, and this leads to the degeneration of both proteins and lipids. The indirect effects arise from the radiation-induced radiolysis of water. Various ions and radicals, such as H_2_O^+^, H^•^, OH^•^, OH^−^, O_2_^•^-, can be formed through the splitting of H_2_O or O_2_ molecules. In turn, these ions and radicals, also called reactive oxygen species (ROS), mainly lead to the same damages as the direct effects in macromolecules like DNA, proteins, and lipids (Riley 1994, Webb and DiRuggiero 2012, Sharma et al. 2017). Since vegetative cells consist of about 80% water and mostly live in an aqueous environment, this plays a significant role in cell damage processes. Microorganisms, like all other living organisms, have evolved various defense mechanisms to counteract ROS-induced damage and to react on oxidative stress.

One key way to survive ionizing radiation exposure is to prevent or repair ROS-induced cellular damage (Sadowska-Bartosz and Bartosz 2023). For example, by producing antioxidant enzymes such as superoxide dismutase (SOD), catalase, and peroxidases. These enzymes catalyze, for example, the conversion of ROS to hydrogen peroxide (H_2_O_2_), which is then split into less harmful molecules, thus neutralizing the damaging effects of ROS (Imlay 2008).

Another strategy is the expression of non-enzymatic antioxidants. Thereby, the cells synthesize or acquire these antioxidants like glutathione, thioredoxin, vitamins, carotenoids, and flavonoids. These molecules scavenge ROS directly, preventing oxidative damage to cellular components (Brynildsen and Liao 2009).

Repair mechanisms are also an option to correct damages caused by ROS. For instance, DNA repair enzymes can correct ROS-induced DNA damage to maintain genomic integrity (Daly et al. 2007).

Alternatively, cells counteract via metal ion homeostasis. Here, the intracellular concentration of free metal ions (e.g. manganese; Mn and iron; Fe) is upregulated, which can counteract ROS-mediated reactions; here, the Mn-concentration is increased in particular (Anjem and Imlay 2012). This theory is based on the observation that the intracellular Mn/Fe-ratio correlates with viability following irradiation based on some examples of radiation-resistant and radiation-sensitive organisms like, e.g. *Deinococcus radiodurans* and *Escherichia coli*, respectively (Daly et al. 2007, Ujaoney et al. 2024). While this theory was based mainly on evidence from experiments with prokaryotic organisms, there have only been limited experiments using Eukarya.

The focus of this study is the investigation of the intracellular Mn/Fe-ratio in 21 organisms of all three domains, e.g. Bacteria, Archaea, and Eukaryotes (yeasts), including eight strict anaerobic and microaerophilic microorganisms never investigated in depth before. Some model organisms were selected based on prior studies in which their radiation tolerance had already been characterized in detail (Beblo et al. 2011). Here, the Mn/Fe-ratio was compared to new and already existing data about the tolerance to ionizing radiation of these strains.

## Material and methods

### List of investigated microorganisms

The microorganisms used in this study are listed in Table 1. They were either grown in large-scale biofermenters at fermentation facilities [Biotechnikum, Archaea-Center, Regensburg, Germany; indicated as (**F**) in Table 1] or cultivated in the laboratories of the German Aerospace Center [DLR, Cologne, Germany; indicated as (**L**) in Table 1]. Strains exhibiting low growth densities were cultivated in large-scale fermenters to obtain sufficient biomass for Mn and Fe analysis. Instead, strains with higher growth densities were grown in large-scale laboratory glass vessels. The final harvested biomass amounted to ~1 g (wet weight) of cells. All strains were grown under their optimal conditions in regard to temperature, pH, salinity, and availability of O_2_, with supplements added as specified in Table 1.

**Table 1:** Cultivated and investigated strains for this study.

Organism, strain, reference	Form of cultivation: fermentation (F) or laboratory (L) and reference for media	Optimal growth temperature	pH during cultivation	Additives (% w/v)	Gas phase (pressure; % v/v) during cultivation for this study	O_2_-metabolism	Origin; phylum
**Archaea**							
*Archaeoglobus fulgidus*, DSM 4304^T^, (Stetter 1988)	**F**, (Huber et al. 1982)	80°C	6.8	0.1% lactate 0.05% YE	H_2_/CO_2_ (300 kPa; 80:20)	Strict anaerobic	Shallow hydrothermal system; Thermoproteota
*Ignicoccus hospitalis*, DSM 18386^T^, (Paper et al. 2007)	**F**, (Blöchl et al. 1997)	90°C	6.0	0.5% S° 0.1% YE	H_2_/CO_2_ (300 kPa; 80:20)	Strict anaerobic	Deep-sea hydrothermal system; Thermoproteota
*Metallosphaera sedula*, DSM 5348^T^, (Huber et al. 1989)	**F**, (Allen 1959), mod. (Brock et al. 1972)	70°C	2.0	0.02% YE	O_2_/CO_2_ (140 kPa; 95:5)	Facultative anaerobic	Hot pond in solfataric field; Thermoproteota
*Methanocaldococcus jannaschii*, DSM 2661^T^, (Jones et al. 1983)	**F**, (Smollett et al. 2017)	85°C	6.3	-	H_2_/CO_2_ (300 kPa; 80:20)	Strict anaerobic	Deep-sea hydrothermal system; Euryarchaeota
*Methanothermobacter thermoautotrophicus*, DSM 1053^T^, (Zeikus and Wolee 1972)	**F, (**Balch et al. 1979)	65°C	7.5	-	H_2_/CO_2_ (300 kPa; 80:20)	Strict anaerobic	Hot spring sediments; Euryarchaeota
*Pyrococcus furiosus*, DSM 3638^T^, (Fiala and Stetter 1986)	**F, (**Blöchl et al. 1997)	95°C	8.0	0.1% starch 0.1% YE 0.1% peptone	N_2_/CO_2_ (300 kPa; 80:20)	Strict anaerobic	Deep-sea hydrothermal system; Thermoproteota
*Sulfuracidifex metallicus*, DSM 6482^T^, (Huber and Stetter 1991)	**F**, (Allen 1959)mod. (Brock et al. 1972)	65°C	2.0	0.1% S° 0.02% YE	O_2_/CO_2_ (200 kPa; 90:10)	Facultative anaerobic	Hot pond in solfataric field; Thermoproteota
*Saccharolobus solfataricus* P8, (Zillig et al. 1980)	**F**, (Allen 1959), mod. (Brock et al. 1972)	80°C	2.0	0.1% S° 0.02% YE	O_2_/CO_2_ (200 kPa; 90:10)	Facultative anaerobic	Hot pond in solfataric field; Thermoproteota
*Thermoproteus tenax* S2, (Zillig et al. 1981)	**F**, (Allen 1959), mod. (Brock et al. 1972)	85°C	6.0	-	N_2_/CO_2_ (300 kPa; 80:20)	Strict anaerobic	Mud hole in solfataric field; Thermoproteota
**Bacteria**							
*Aquifex pyrophilus*, DSM 6848^T^, (Huber et al. 1992)	**F**, (Huber et al. 1992)	85°C	6.8	0.5% S°	N_2_/CO_2_/O_2_ (300 kPa; 79.75:19.75:0.5)	Microaerobic	Deep-sea hydrothermal system; Aquificota
*Buttiauxella* sp. MASE-IM-9, DSM 105071, (Cockell et al. 2018)	**L**, Tryptic soy broth No. 2	30°C	7.0	-	Ambient air	Facultative anaerobic	Sediment sample sulfidic stream; Proteobacteria
*Chromohalobacter sarecensis*, DSM 15547, (Quillaguaman et al. 2004)	**L**, Marine broth 2216	30°C	7.7	6% NaCl	Ambient air	Strict aerobic	Saline soil around lake; Proteobacteria
*Deinococcus radiodurans*, DSM 20539^T^, (Anderson et al. 1956)	**L**, Tryptone-Glucose-Yeast medium	30°C	7.0	-	Ambient air	Strict aerobic	Irradiated can; Deinococcota
*Escherichia coli*, DSM 18039, (Jensen 1993)	**L**, Luria-Bertani medium	37°C	7.0	-	Ambient air	Facultative anaerobic	Origin unknown; Proteobacteria
*Hydrogenothermus marinus*, DSM 12046^T^, (Stohr et al. 2001)	**F**, mod. (ZoBell 1941)	65°C	6.5	4% NaCl	H_2_/CO_2_/O_2_ (200 kPa; 78:20:2)	Microaerobic	Marine hydrothermal system; Aquificota
*Paenisporosarcina antarctica*, DSM 21991, (Reddy et al. 2013), (Yu et al. 2008)	**L**, Marine broth 2216	18°C	7.0	-	Ambient air	Strict aerobic	Sediment Antarctica; Bacillota
*Planococcus halocryophilus*, DSM 24743, (Mykytczuk et al. 2012)	**L**, Tryptic soy broth No. 2	25°C	7.5	-	Ambient air	Strict aerobic	Permafrost sediment; Bacillota
*Salinisphaera shabanensis*, DSM 14853^T^, (Antunes et al. 2003)	**L**, Marine broth 2216	30°C	7.0	10% NaCl	Ambient air	Strict aerobic	Deep-sea brine–seawater interface; Pseudomonadota
*Staphylococcus capitis*, DSM 111179, (Sobisch et al. 2019)	**L**, Tryptic soy broth No. 2	37°C	7.0	-	Ambient air	Strict aerobic	Surface inside of ISS; Bacillota
**Eukarya**							
*Rhodotorula frigidalcoholis* EXF-10854, formerly JG1b, (Touchette et al. 2022)	**L**, Universal medium for yeast (DSM 186)	25°C	7.0	-	Ambient air	Strict aerobic	Permafrost sediment Antarctica; Basidiomycota
*Rhodotorula mucilaginosa*, DSM 114276, (Moore and Breedveld 1989)	**L**, Universal medium for yeast (DSM 186)	25°C	7.0	-	Ambient air	Strict aerobic	Surface inside of cleanroom; Basidiomycota

^T^ refers to type strain. Strains were cultivated under optimal conditions in terms of temperature, pH, additives, and oxygen availability. Additionally, the O2-metabolism and the origin of the strains is shown.

DSM: number of the strain in the catalogue of German Collection of Microorganisms and Cell Cultures GmbH (DSMZ), YE: yeast extract, S°: elemental sulphur, ISS: International Space Station.

**Table 2:** 6 Mn/Fe-ratio calculated from the quantitative determination of metals by either ICP-MS or ICP-OES and *D*_10_-values measured in this study (indicated in bold) and collected from literature.

Archaea		
Organism (references where applicable)	Mn/Fe-ratio	*D*_10-_value (Gy)
*Archaoglobus fulgidus* (Beblo et al. 2011)	**0.03296 ± 0.003165**	1090
*Halobacterium salinarum* (Kish et al. 2009)	0.27	5000
*Haloferax volcanii* (Kottemann et al. 2005, Kish et al. 2009, Sharma et al. 2017)	0.28	1500
*Ignicoccus hospitalis* (Koschnitzki et al. 2021)	**0.00059 ± 0.000505**	4700
*Metallosphaera sedula* (Beblo et al. 2011)	**0.00007** ^**^#^**^	490
*Methanocaldococcus jannaschii* (Beblo et al. 2011)	**0.00275 ± 0.000222**	1040
*Methanothermobacter thermautotrophicus*	**0.73377 ± 0.040578**	**2750**
*Pyrococcus furiosus* (Webb and DiRuggiero 2012)	0.04	3000
*Ppyrococcus furiosus* DLR (Beblo et al. 2011)	**0.03397 ± 0.013189**	1020
*Saccharolobus solfataricus* (Beblo et al. 2011)	**0.00001 ± 0.000004**	1020
*Sulfuracidifex metallicus* (Beblo et al. 2011)	**0.04686 ± 0.004601**	760
*Thermococcus gammatolerans* (Webb and DiRuggiero 2012)	0.01	6000
*Thermoproteus tenax* (Beblo et al. 2011)	**0.00518 ± 0.000249**	870
**Bacteria**		
*Acinetobacter radioresistens* (Sharma et al. 2017)	0.15	5000
Alphabroteobacterium 4A6 (Daly 2009)	0.15	1500
*Aquifex pyrophilus* (Beblo et al. 2011)	**0.01129 ± 0.001363**	2840
*Bacillus subtilis* spores (Granger et al. 2011)	0.3	2000
*Bacillus anthracis* spores (Tu et al. 2012, Cote et al. 2018)	0.41	2300
*Buttiauxella* sp. MASE-IM-9 (Beblo-Vranesevic et al. 2018)	**0.04902 ± 0.001053**	500
*Deinococcus deserti* (De Groot et al. 2005, 2009)	0.54	5250
*Deinococcus ficus* (Matrosova et al. 2017, Sharma et al. 2017)	0.46	7000
*Deinococcus geothermalis* (Daly et al. 2004)	0.46	10 000
*Deinococcus radiodurans* 7b-1 (Fredrickson et al. 2008, Daly 2009)	0.14	13 000
*Deinococcus radiodurans* R1 (Daly et al. 2004)	0.24	16 000
*Deinococcus radiodurans* DLR (Bauermeister et al. 2011)	**0.31408 ± 0.044003**	5500
*Deinococcus* sp. 1A1 (Daly 2009)	0.15	17 000
*Deinococcus* sp. 5A5 (Daly 2009)	0.38	15 000
*Deinococcus* sp. 1A6 (Daly 2009)	0.15	7000
*Deinococcus* sp. 3B1 (Daly 2009)	0.12	10 500
*Enterococcus faecium* (Daly et al. 2004)	0.17	2000
*Escherichia coli* (Daly et al. 2004)	0.0072	700
*Escherichia coli* DLR (Trampuz et al. 2006)	**0.05604 ± 0.036069**	310
*Hydrogenothermus marinus* (Beblo et al. 2011)	**0.03084 ± 0.001271**	750
*Kineococcus radiotolerans* (Bagwell et al. 2008a, 2008b)	0.087	6000
*Macrococcus caseolyticus* (Karani et al. 2015)	0.027	607
*Neisseria gonorrhoeae* (Daly 2009)	0.004	125
*Paenisporosarcina antarctica*	**0.00075 ± 0.000007**	**520**
*Planococcus halocryophilus*	**0.17458 ± 0.026111**	**510**
*Pseudomonas putida* (Daly et al. 2004, Robinson et al. 2011)	0.017	250
*Rubrobacter radiotolerans* (Ferreira et al. 1999, Webb and DiRuggiero 2012)	0.88	12 000
*Rubrobacter xylanophilus* (Ferreira et al. 1999, Webb and DiRuggiero 2012)	1.9	6000
*Salinisphaera shabanensis*	**0.00248 ± 0.000028**	**1700**
*Shewanella oneidensis* (Daly et al. 2004)	0.0005	70
*Staphylococcus aureus* (Karani et al. 2012)	0.009	194
*Staphylococcus capitis* (Siems et al. 2022)	**0.31472 ± 0.008334**	98
*Thermus thermophilus* (Omelchenko et al. 2005)	0.047	800
**Eukarya**		
*Chlorella* sp. (Tokuşoglu and Üunal 2003, Park and Choi 2018)	0.008	9170
*Saccharomyces cerevisiae* (Groombridge et al. 2013, Diessl et al. 2022)	0.138	1000
*Rhodotorula frigidialcoholis*	**0.04826 ± 0.003919**	**640**
*Rhodotorula mucilaginosa*	**0.03667 ± 0.000667**	**680**

# No standard deviation is shown because the Mn concentration was below detection limit in two replicates. Detailed and absolute numbers of Mn and Fe related to cell mass, protein content, and cell numbers can be found in Supplementary Tables 1–3.

### Cell harvest and sample preparation

Cells were cultivated under the indicated conditions (Table 1). The cells in the large-scale fermentation facilities (up to 300 l) were grown to the stationary growth phase and harvested overnight by centrifugation. The cell pellets were flash-frozen in liquid nitrogen and stored at −150°C. Approximately 2 g of frozen samples were delivered to DLR for further investigations and stored at −80°C. To prepare samples from frozen biomass, the samples were thawed at 4°C overnight. Aliquots (300 µl) were transferred into 5 ml of adapted phosphate-buffered saline (PBS; 4.0 g NaCl, 3.0 g KH₂PO₄, 7.0 g Na₂HPO₄ per liter H₂O, adjusted to pH 7.4), which served as a washing buffer solution. PBS was adapted in terms of salinity (NaCl) and pH to the conditions of the respective strain as indicated in Table 1. To wash the cells, samples were mixed until fully re-suspended, centrifuged, and the supernatant carefully removed.

To prepare samples from fresh cultures, 800 ml of each liquid medium was inoculated and incubated until the stationary growth phase was reached. Cells were harvested by cycles of centrifugation and supernatant removal. The pellets were then resuspended in adapted PBS to wash the biomass. All centrifugation steps were performed at 4500 g for 25 min at 2°C.

From this point onward, all samples collected from fresh and frozen biomass were processed in the same manner. Cell pellets were obtained again by centrifugation and supernatant removal. The wet weight of the biomass samples was recorded, and samples were stored at 4°C. All supernatant fractions from every sample were removed after each centrifugation step and stored for further analysis.

### Cell numbers and determination of protein content

To determine reference parameters, cell pellets were resuspended in adapted PBS, and a sub-sample of the cell suspension was taken to determine the total cell number (Thoma cell count chamber; depth: 0.02 mm) and protein content. The protein content was measured after cell lysis using the Bradford assay. For each sample, 200 µl aliquots were taken, and B-PER™ (Bacterial Protein Extraction Reagent; Thermo Fisher Scientific) was added to disrupt the cells according to the manufacturer’s protocol, for prokaryotic and archaeal organisms. Eukaryotic cells were lysed by heat shock and bead beating. The protein concentration was then determined using the Pierce™ Coomassie (Bradford) Protein Assay Kit (Thermo Fisher Scientific), following the manufacturer’s instructions.

### Exposure to ionizing radiation and determination of the survival fraction

To determine *D*_10_-values of the organisms, cells were treated with X-rays in-house as described earlier (Beblo et al. 2011). Briefly, liquid cell cultures, with a density between 10^7^ and10^8^ cells per ml, were irradiated in 2 ml plastic tubes (*Buttiauxella* sp. MASE-IM-9, *C. sarecensis, D. radiodurans, E. coli, P. antarctica, P. halocryophilus, S. shabanensis, S. capitis, R. frigidalcoholis, R. mucilaginosa*) or in 4 ml glass vessels (all other organisms). Irradiation was carried out with the X-ray source Gulmay RS 225A (Gulmay Medical, Ltd.) at 200 kV and 15 mA without a filter. The cells were irradiated at a distance of 19.5 cm below the X-ray source with 20 ± 5 Gy/min. The dose rate was measured with a UNIDOS dosimeter (PTW Freiburg GmbH). All irradiation experiments were performed at room temperature. All anaerobic and microaerophilic organisms were kept always under anoxic conditions, including the ionizing radiation treatment (see O_2_-metabolism in Table 1).

For the determination of the survival rate, aliquots were taken under anoxic or oxic conditions, and the survival was determined via the most probable number (MPN) technique in dilutions series (for organisms not able to grow on solid media: *A. fulgidus, I. hospitalis, M. sedula, M. jannaschii, M. thermoautotrophicus, P. furiosus, S. metallicus, S. solfataricus, T. tenax, A. pyrophilus, H. marinus*) or via plating assay on agar plates (all other organisms). For *Buttiauxella* sp., MASE-IM-9 and *S. shabanensis* previous experiments showed that the results of MPN-technique and plating assay are comparable (Beblo-Vranesevic et al. 2018, 2020, 2022).

The irradiation experiments were conducted at least in triplicates through three independent experiments.

The survival rate (*S*) was calculated as relative survival after cell-damaging treatment (*N*) compared to the non-treated control (*N*_0_), also referred to as the dark control (*S* = *N*/*N*₀). This normalization allows comparison of survival independently of initial cell density. *D*_10_-values were determined from survival curves following irradiation and represent the dose (Gy) required to reduce survival by one order of magnitude. The values were calculated from the regression lines of the exponential slopes of the survival curves (Harm 1980, Peleg 2023).

### Analysis of Fe and Mn concentration

Metal contents of the samples, supernatant, adapted PBS buffer, and media were determined at the Elements and Minerals of the Earth Laboratory at GFZ Potsdam. Samples were treated with 100 µl concentrated (69%–70%) nitric acid at 95°C for 2 h. Hydrogen peroxide was then added to a final concentration of 5 mM, and samples were further incubated for 90 min at 75 °C. The digested samples were diluted using ultra-pure reagents and deionized water prior to analysis. The metal content of each digested sample was determined by high-resolution inductively coupled plasma mass spectrometry (HR-ICP-MS; Element XR series 2, Thermo Fisher Scientific) or by ICP-OES (5110, Agilent). Concentrations were determined by external calibration for both instruments. For ICP-MS, an internal standard was used to correct for instrumental mass bias. The results obtained from both methods are comparable.

The measurement of intracellular Mn and Fe concentrations was performed in at least triplicate.

### Data treatment and calculations of intracellular Mn/Fe-ratio

The concentrations of Mn and Fe were measured in the pellets, as well as in the supernatant after washing and in the washing buffers as controls. The final amount of Mn and Fe present in the sample, corresponding to the intracellular amount of Mn and Fe, was determined by subtracting the amount found in the supernatant from that in the biomass.

The absolute measured quantities of Mn and Fe were normalized to cell numbers and protein content, respectively, and their ratio was determined. This intracellular Mn/Fe-ratio was then correlated with the *D*_10_-value. Both the Mn/Fe-ratio and the *D*_10_-value were plotted in a double-logarithmic scale to assess potential dependency, and a regression curve was plotted. The coefficient of determination (*r*^2^) and regression coefficient (*b*[1]) were determined to describe a possible correlation. Thereby, *r*^2^ gives the proportion of the variance of the variables, and the regression coefficient indicates the slope of the regression line. In the case of a maximum dependency, *b*[1] would be equal to 1.

## Results

### Mn/Fe-ratio and *D*_10_-value of investigated organisms and comparison with literature data

The focus of this study was to determine whether the Mn/Fe-ratio could explain radiation tolerance; when *D*_10_-values were unavailable or unknown, in-house laboratory data were used or *D*_10_-values were experimentally determined. To address this objective, intracellular Mn and Fe levels were quantified relative to biomass, total intracellular protein content, and cell number, as presented in Supplementary Tables 1–3. Based on these measurements, Mn/Fe-ratios were calculated, and their potential impact on cell survival following exposure to ionizing radiation was evaluated. Irradiation experiments were conducted using plastic tubes or glass vessels. Previous experiments with *Buttiauxella* sp. MASE-IM-9, *D. radiodurans, E. coli*, and *S. shabanensis* demonstrated no significant difference in survival outcomes between cells irradiated in plastic versus glass containers (data not shown).

**Figure 1:**
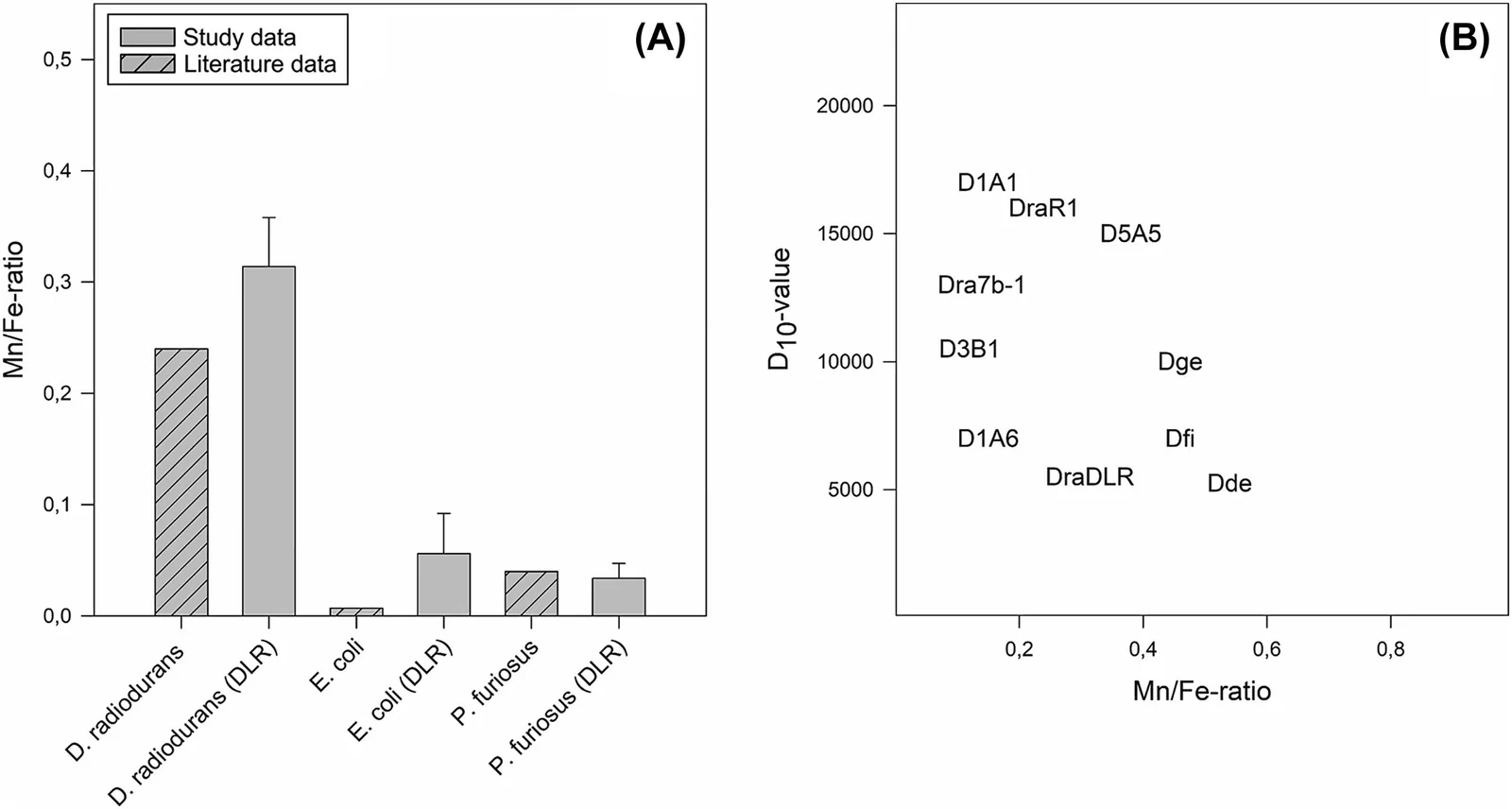
Comparison of measured intracellular Mn/Fe-ratio with literature data. (A) Striped columns correspond to literature data (Table 2). Unstriped columns are values measured in this study in triplicates with standard deviation. (B) Comparison of Mn/Fe-ratio and *D*_10_-value of different *Deinococcus* species. Dde: *Deinococcus deserti*, Dfi: *Deinococcus ficus*, Dge: *Deinococcus geothermalis*, DraR1: *Deinococcus radiodurans* R1, Dra 7b-1: *Deinococcus radiodurans* 7b-1, D1A1: *Deinococcus* sp. 1A1, D5A5: *Deinococcus* sp. 5A5, D1A6: *Deinococcus* sp. 1A6, D3B1: *Deinococcus* sp. 3B1, DraDLR: *Deinococcus radiodurans* DLR.

There is an offset in the Mn/Fe-ratios between the results from this study and those reported in the literature to some extent. The data were within the same range for *P. furiosus* (Fig. 1A), indicating the comparability of the measured data (ICP-OES) with literature data (mostly ICP-MS). At the concentration levels of Mn and Fe measured here, both ICP-OES and ICP-MS provide reliable and directly comparable results. However, the values obtained in this study were higher for *D. radiodurans* and *E. coli*. The possible reasons that could explain these differences include different growth phases, growth conditions (e.g. medium), and strains (in the case of *E. coli*), which will be further discussed later.

All *Deinococcus* species cluster together, with the measured data of *D. radiodurans* DLR (DraDLR) included, indicating their similarity in measured values (Fig. 1B).

### Dependence between Mn/Fe-ratio, *D*_10_-value, and domain in measured data and comparison with literature data

**Figure 2:**
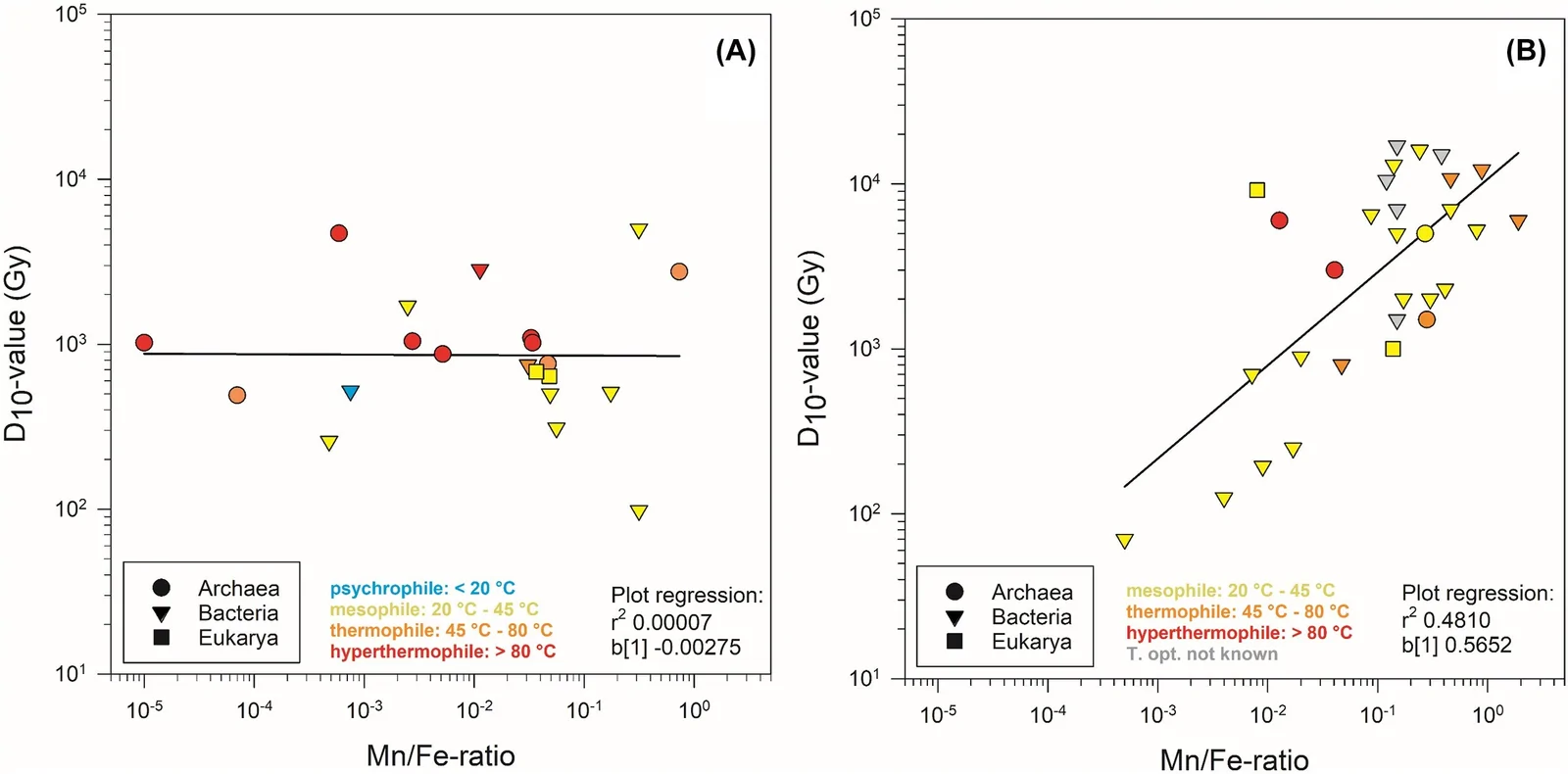
Double logarithmic representation of *D*_10_-value and Mn/Fe-ratio in the same scaling in both figures. All data originating from Table 2. Archaea are symbolized as circles, Bacteria as triangles, and Eukarya as squares. The different colors represent the organisms’ optimal growth temperature. Regression lines and regression analyses are based on all data in plot. (A) Measured Mn/Fe-ratio, *D*_10_-value and domain of investigated microorganisms in this study. (B) Visualization of Mn/Fe-ratio and *D*_10_-value from literature data.

The calculated Mn/Fe-ratios with respect to the *D*_10_-values are distributed throughout the entire graph in a double logarithmic fashion (Fig. 2A). No distinct dependence on the domain of life the organism under study belongs to can be observed. The two eukaryotic representatives are closely grouped, indicating their close evolutionary relationship, both belonging to *Rhodotorula*. There is no specific range that is solely dominated by Archaea or Bacteria. A closer look at the data and considering the optimum growth conditions, like temperature or pH, does not reveal any dependence on these parameters either (Fig. 2A). When examining the literature data (Mn/Fe-ratio and *D*_10_-value) using the same representation as in the measured data, a dependence between the Mn/Fe-ratio and the *D*_10_-value can be discerned (Fig. 2B). This general difference between measured data and data from literature is supported by the plot regression values (measured: *r*^2^ 0.00007, *b*[1] −0.00275 vs. literature data: *r*^2^ 0.4810, *b*[1] 0.5652).

## Discussion

In addition to the direct radiation effect where radiation has direct impacts on DNA integrity (single and double strand breaks), indirect radiation effect affects interaction with other molecules (e.g. water molecules) in the cell to produce free radicals, among them ROS, which in turn induce cellular damage. Tolerance to ionizing radiation, and consequently the tolerance to ROS, is a common phenomenon across all domains of life (Beblo et al. 2011, Beblo-Vranesevic et al. 2017, 2018). However, the reasons for these tolerances remain to be determined. It is suggested that these tolerances are a multifactorial trait involving small RNAs, the cultivation conditions, the Mn and Fe homeostasis, enzyme-based protection mechanisms (e.g. SODs), the metabolism (O_2_), the growth temperature, and specialized DNA repair systems (Ujaoney et al. 2024). These observations align with the emerging consensus that Mn/Fe-ratios are important but not solely determinative, and other molecular and environmental factors substantially influence radiation tolerance (Sweet et al. 2024).

Recent studies indicate that small regulatory RNAs, such as DrsS in *D. radiodurans*, are induced by ionizing radiation and actively contribute to the detoxification of ROS, complementing classical antioxidant systems (Rai and Dutta 2024). Although small regulatory RNAs have been identified in some organisms tested here, their roles in radiation response or ROS detoxification remain unclear (Wagner and Romby 2015).

Measurements of Mn and Fe represent only a snapshot of the cellular homeostatic state. Differences in cultivation conditions can influence intracellular Mn and Fe accumulation and may therefore explain the variability observed in Mn/Fe-levels. For *D. radiodurans*, it has been shown that different growth media result in distinct intracellular Mn/Fe-ratios (Daly et al. 2004). In addition, Mn/Fe-levels and tolerance to ionizing radiation in *D. radiodurans* vary with the growth phase (Sukhi et al. 2009). Such factors may also contribute to the reported differences in Mn/Fe-levels and survivability following ionizing radiation treatment, for example, in *D. radiodurans*, where *D*_10_-values ranging from 5.5 to 16 kGy have been reported (Daly et al. 2004, Bauermeister et al. 2011). Daly et al. (2004) determined a Mn/Fe-value of 0.24 in *D. radiodurans* R1 vs. 0.31 measured in *D. radiodurans* R1 DLR in this study.

Mn and Fe ions play a crucial role in the tolerance to ionizing radiation, as has been demonstrated in some microorganisms, among them again the well-known model organism *D. radiodurans*. However, our results do not support a simple or consistent correlation between the tolerance to ionizing radiation and the intracellular Mn/Fe-ratio.

ROS are not only produced during exposure to ionizing radiation, but also occur naturally in lower amounts during aerobic and anaerobic metabolism or can even be available in the environmental habitat of the organisms (Fee 1982, Imlay 2019). Therefore, all organisms need to have defense mechanisms against ROS and oxidative stress. One key defense mechanism is the presence of proteins containing a metallic ion. These metals serve as co-factors to the enzymes they are bound to, playing crucial roles in catalyzing biochemical reactions (Cannio et al. 2000). Detoxification examples include SOD alone or in combination with a catalase system. Superoxide reductases, or SOD, containing metal cofactors such as manganese, iron, nickel, or copper/zinc combinations, recognize ROS such as superoxides (O_2_^−^) and hydroxyl radicals (·OH) and convert them to H_2_O_2_. Catalases and hydroperoxide reductases subsequently convert H_2_O_2_ into water and oxygen (Seaver and Imlay 2001, Imlay 2008). Catalase enzymes often contain iron or manganese at their active sites (Yuan et al. 2021). Other examples include manganese-dependent peroxidases, which require Mn ions for catalytic activity (Singh et al. 2013). Recent research highlights that the SOD/catalase system remains central for ROS detoxification, but additional protective mechanisms include Mn-antioxidant complexes and radiation-induced peptides (Pedone et al. 2020, Rai and Dutta 2024, Yang et al. 2024).

In *Aquifex aeolicus*, and probably also in the investigated close relative *A. pyrophilus*, three SOD genes are present, but catalases are absent (Brugna-Guiral et al. 2003). Nevertheless, several peroxidases, including a cytochrome c peroxidase and a hydroperoxide reductase, have been identified at the genome level, which could take over the removal of H_2_O_2_ (Deckert et al. 1998). In *A. pyrophilus*, a thermostable Fe-SOD has been described and characterized (He et al. 2007). *Archaeoglobus fulgidus* possesses a catalase/peroxidase system (Klenk et al. 1997) and *P. furiosus* a superoxide reductase/peroxidase system (Jenney et al. 1999), which was shown to be induced by treatment with ionizing radiation (Williams et al. 2007), and both organisms react in the same way to ionizing radiation documented by a nearly same *D*_10_-value around 1000 Gy (Beblo et al. 2011). Also, *M. thermoautotrophicus* possesses an Fe-SOD (Takao et al. 1990), and *I. hospitalis* has a superoxide reductase and cytoplasmic peroxiredoxin system (Burghardt et al. 2008, Podar et al. 2008). Nevertheless, both Archaea have significantly different *D*_10_-values (*M. thermoautotrophicus*: 2750 Gy vs. *I. hospitalis*: 4700 Gy) (Beblo et al. 2011). In *E. coli*, both Mn- and Fe-SOD have been identified (Beyer et al. 1991). *Rhodotorula* species also possess Fe- and Mn-SODs, providing protection against oxidative stress (Kan et al. 2017). Additionally, Cu/Zn-SOD genes are present but often not expressed under normal conditions (Mosqueda-Martínez et al. 2024).

Mn ions alone can scavenge ROS, contributing to radiation tolerance, though higher intracellular Mn levels are required than for SOD-mediated protection (Daly 2012, McKeen 2012, Webb and DiRuggiero 2012). For instance, *D. radiodurans* contains high concentrations of free Mn ions and Mn-antioxidant complexes (Sharma et al. 2017). Interestingly, some organisms with low Mn levels still survive irradiation well (e.g. Sulfolobales strains: *M. sedula, S. solfataricus*), showing that Mn/Fe-ratios alone cannot fully explain radiation tolerance.

Despite not being measured in this study, pigments can also act as non-enzymatic antioxidants (non-containing Mn or Fe ions), and some of the tested microorganisms may possess these due to their morphologically colorful colony formations. These include the bacteria *C. sarecensis, D. radiodurans, P. antarctica, P. halocryophilus, S. capitis*, as well as the yeasts *R. frigidalcoholis* and *R. mucilaginosa*. Under oxic conditions, organisms tend to favor Fe in contrast to Mn as a cofactor for aerobic metabolism (Daly 2012). Free Fe can participate in redox reactions but also catalyze ROS formation via the Fenton reaction (Figueiredo et al. 2012). In anoxic environments, Mn-dependent enzymes, such as certain SODs, may become more prevalent, potentially increasing the intracellular Mn/Fe-ratio (Jakubovics and Jenkinson 2001). However, tested anaerobic organisms do not show a strict correlation between Mn/Fe-ratios and radiation tolerance, suggesting that additional protective factors, such as antioxidants and DNA repair systems, are involved. During irradiation, extracellular and intracellular oxygen can be radiolyzed into ROS, making oxygen a radiation sensitizer (Kiefer 2013). Reduced oxygen in the irradiation medium could decrease radiation damage (Michaels et al. 1978). Some of the microorganisms with the highest survival rates in our study (microaerophilic *A. pyrophilus* or strictly anaerobic strains like *I. hospitalis* and *M. thermoautotrophicus*) were irradiated in reducing media, limiting oxygen availability. Reducing agents in the medium (Na_2_S, L-cysteine) may further scavenge ROS, enhancing survival. However, further details on the intra- and extracellular redox state cannot be provided, as no direct measurements are available.

A correlation between growth temperature and radiation tolerance is possible. High temperatures can increase metabolic ROS production and damage cellular components. Membrane rigidity in hyperthermophiles helps prevent penetration of ROS (Cario et al. 2015, Siliakus et al. 2017). Indeed, hyperthermophiles exhibit high *D*_10_-values [*I. hospitalis*: 4700 Gy (Koschnitzki et al. 2021); *M. thermoautotrophicus*: 2750 Gy (Beblo et al. 2011)], although not all thermophiles display uniform responses. Repair systems are critical in these organisms to counteract the adverse effects of heat and radiation. Many of the microorganisms investigated in this study encode either RecA or its archaeal homolog RadA, indicating that homologous recombination systems are broadly conserved across diverse lineages (Del Val et al. 2019, Hogrel et al. 2020). RadA/RecA is vital for repairing radiation- and radical-induced damage (Carlsson and Carpenter 1980, Zhou et al. 2006). However, the regulatory dynamics and functional efficiency of these systems vary substantially between species, contributing to the wide range of observed radiation tolerances (Haldenby et al. 2009). The presence of RecA/RadA system alone does not predict tolerance, highlighting the complex and multifactorial nature of radioresistance (Rai and Dutta 2024, Sweet et al. 2024).

In conclusion, tolerance to ionizing radiation is widely distributed across the tree of life, yet its evolutionary origins remain incompletely understood (Daly 2023, Shukla et al. 2024). This study analyzed prokaryotes and two eukaryotic yeast strains, integrating data with recent literature to underscore the diversity and specificity of radiation resistance. The Mn/Fe-SOD family, which is conserved across Bacteria, Archaea, and Eukarya and likely derived from a common ancestor over 3 billion years ago (Aguirre and Culotta 2012), highlights the fundamental importance of ROS-defense systems in evolution. Recent work further emphasizes how radiation tolerance is strain-specific and dynamically regulated: novel non-sporulating bacterial isolates with unexpectedly high radiation tolerance have been identified, with whole-genome sequencing revealing conserved DNA repair systems such as RecA and UvrABC (Cassilly et al. 2025), and halophilic Archaea have been shown to exhibit dramatically different X-ray sensitivities even within the same lineage (Runzheimer and Leuko 2025). Extracellular vesicles derived from *D. radiodurans* have been demonstrated to provide radioprotective effects in mammalian tissues (Han et al. 2025). Taken together, these findings strongly support the notion that tolerance to ROS—including that elicited by ionizing radiation—is not a monolithic trait but a highly strain-specific, evolutionarily tuned adaptation.

However, several limitations should be considered when interpreting the present dataset. Methodological heterogeneity among experiments, including differences in IPC approaches (ICP-MS versus ICO-OES) and survival assessment methods (MPN technique versus plating assay), may reduce direct comparability of results. In addition, variations in cultivation conditions, including temperature and growth phase, likely influenced the measured responses; ROS-scavenging reducing conditions in the growth medium could have contributed to these effects. The dataset also includes only a limited number of eukaryotic organisms and lacks broader representation of cold-adapted strains. Furthermore, some observations may reflect strain-specific rather than genus-wide characteristics. Future studies should therefore apply standardized methods, expand taxonomic diversity, include more samples across varied cultivation conditions, and directly compare multiple strains within the same genus to disentangle lineage effects from strain-level variation. A clearer understanding of how the investigated defense mechanisms against ionizing radiation diversify and function will be valuable for biotechnology, medicine, and astrobiology.

## Supplementary Material

xtag029_Supplemental_Files
