# Supplementary material for: Elucidating the interplay between intracellular manganese–iron-ratio and radiotolerance across all domains of life
Source: FEMS Microbes. 2026 May 28;7:xtag029. doi: 10.1093/femsmc/xtag029 (PMC13251893; doi:10.1093/femsmc/xtag029)
Supplement: xtag029_Supplemental_Files [file xtag029_supplemental_files.zip › Supplementary_final.docx]

**Supplementary materia**l

Quantitative determination of metals (Mn and Fe) by ICP-MS

Supplementary Table 1. Intracellular Mn and Fe values normalized to biomass amount, protein content and total cell number of Archaea. *No standard deviation because Mn below detection limit in two replicates.

| **Archaea** | | | | | | |
| --- | --- | --- | --- | --- | --- | --- |
| **Organism** | **µg Mn/g biomass (Standard deviation)** | **µg Fe/g biomass (Standard deviation)** | **nmol Mn/mg protein (Standard deviation)** | **nmol Fe/mg protein (Standard deviation)** | **ng Mn/10^9^ cells (Standard deviation)** | **ng Fe/10^9^ cells (Standard deviation)** |
| *A. fulgidus* | 25.8 (7.4) | 800 (281.5) | 3.2 (0.8) | 96.4 (31.4) | 487 (34.6) | 14917 (2380) |
| *I. hospitalis* | 0.12 (0.16) | 140 (84.1) | 0.06 (0.08) | 86.4 (28.2) | 0.95 (1.34) | 1118 (682) |
| *M. sedula* | 0.14* | 1642 (354) | 0.16* | 6949 (6818) | 0.28* | 3163 (1097) |
| *M. jannaschii* | 1.4 (0.4) | 508 (145) | 1.4 (1.3) | 475.12 (436.17) | 9.2 (0.7) | 3350 (134) |
| *M. thermautotrophicus* | 859 (257) | 1162 (295) | 18074 (8243) | 24667 (12701) | 1833 (541) | 2477 (616) |
| *P. furiosus* | 22.5 (9.1) | 667.3 (96.5) | 80.8 (82.3) | 2123 (1698) | 53.8 (17.3) | 2005 (1631) |
| *S. solfataricus* | 0.11 (0.03) | 7974 (315) | 0.39 (0.06) | 39204 (21543) | 0.17 (0) | 13543 (3014) |
| *S. metallicus* | 5.6 (0.9) | 121 (22.7) | 27.1 (19.0) | 573 (388) | 12.7 (1.4) | 272 (31.8) |
| *T. tenax* | 2.18 (0.24) | 421 (55.0) | 0.43 (0.11) | 82.7 (22.3) | 3.8 (1.6) | 739 (297) |

Supplementary Table 1. Intracellular Mn and Fe values normalized to biomass amount, protein content and total cell number of Archaea. *No standard deviation because Mn below detection limit in two replicates.

Supplementary Table 2. Intracellular Mn and Fe values normalized to biomass amount, protein content and total cell number of Bacteria.

| **Bacteria** | | | | | | |
| --- | --- | --- | --- | --- | --- | --- |
| **Organism** | **µg Mn/g biomass (Standard deviation)** | **µg Fe/g biomass (Standard deviation)** | **nmol Mn/mg protein (Standard deviation)** | **nmol Fe/mg protein (Standard deviation)** | **ng Mn/10^9^ cells (Standard deviation)** | **ng Fe/10^9^ cells (Standard deviation)** |
| *A. pyrophilus* | 5.4 (1.1) | 477 (76.8) | 1.11 (0.29) | 96.9 (21.4) | 11.5 (5.6) | 1010 (449) |
| *Buttiauxella sp.* MASE-IM-9 | 2.4 (0.05) | 49 (0.82) | 0.32 (0.02) | 6.5 (0.59) | 1.3 (0.08) | 26 (2.2) |
| *C. sarecensis* | 4.7 (0.12) | 9830 (304) | 5.2 (1.8) | 10710 (3546) | 122 (51.9) | 255719 (109207) |
| *D. radiodurans* | 4.8 (4.32) | 15.4 (13.4) | 9.9 (16.4) | 23.4 (25.5) | 106 (58.2) | 354 (208) |
| *E. coli* | 2.4 (1.3) | 45.9 (10.6) | 0.69 (0.34) | 13.6 (6.1) | 9.5 (4.0) | 242 (136) |
| *H. marinus* | 19.2 (0.8) | 621 (23.7) | 0.99 (1.06) | 30.7 (32.4) | 20.7 (6.7) | 671 (210) |
| *P. antarctica* | 2.3 (0.06) | 3036 (83.7) | 49.7 (18.7) | 64890 (24844) | 5.1 (0.74) | 6687 (963) |
| *P. halocryophilus* | 6.5 (0.15) | 37.9 (7.0) | 21.2 (2.3) | 120 (9.1) | 4.5 (0.95) | 25.6 (2.5) |
| *S. shabanensis* | 14.8 (0.22) | 5958 (77.6) | 6.2 (1.2) | 2456 (461) | 7.9 (3.5) | 3186 (1420) |
| *S. capitis* | 13.6 (1.2) | 43.1 (3.1) | 4.6 (1.0) | 14.4 (3.3) | 56.3 (18.7) | 178 (55.4) |

Supplementary Table 3. Intracellular Mn and Fe values normalized with biomass amount, protein content and total cell number of Eucarya.

| **Eukarya** | | | | | | |
| --- | --- | --- | --- | --- | --- | --- |
| **Organism** | **µg Mn/g biomass (Standard deviation)** | **µg Fe/g biomass (Standard deviation)** | **nmol Mn/mg protein (Standard deviation)** | **nmol Fe/mg protein (Standard deviation)** | **ng Mn/10^9^ cells (Standard deviation)** | **ng Fe/10^9^ cells (Standard deviation)** |
| *R. frigidialcoholis* | 0.74 (0.02) | 15.3 (1.19) | 0.43 (0.26) | 9.0 (5.3) | 13.6 (1.0) | 284 (33.9) |
| *R. mucilaginosa* | 0.59 (0.01) | 16.2 (0.44) | 1.99 (1.52) | 53.1 (39.5) | 8.4 (1.6) | 229 (45.5) |
