# Supplementary material for: Elucidating the interplay between intracellular manganese–iron-ratio and radiotolerance across all domains of life
Source: FEMS Microbes. 2026 May 28;7:xtag029. doi: 10.1093/femsmc/xtag029 (PMC13251893; doi:10.1093/femsmc/xtag029)
Supplement: xtag029_Supplemental_Files [file xtag029_supplemental_files.zip › FEMSMC-2025-072.R2 supplementary data - reviews and author response.docx]

**Reviews of manuscript FEMSMC-2025-072**

**Reviewer 1**

**Recommendation: Major Revision**

Beblo-Vranesevic and colleagues address the interesting question about the relationship between radiation tolerance and cellular metal homeostasis across microorganisms from all three domains of life. However, substantial clarification and additional methodological details are needed to improve the transparency and reproducibility of the findings. In particular, the rationale for organism selection, as well as significant differences in culturing approach, counting, and elemental analysis methods across the different strains, is needed. Additionally, further justification is required for integrating the newly generated Mn/Fe data with existing literature D10 values (rather than re-doing those measurements to ensure consistency across the entire dataset). The discussion section would also benefit from a stronger focus on interpreting the study’s own results (especially the discrepancies between the findings here and in the literature) rather than merely reviewing prior work. My detailed comments follow.

Major Comments

-It would be helpful to provide the rationale behind the selection of the organisms listed in Table 1. It would also be helpful to include additional metadata in this table: environment of origin of each strain, phylum (I think the authors may be going for phylogenetic breadth here), whether these are strict aerobes/facultative anaerobes/strict anaerobes (beyond just listing “aerobic” and “anaerobic”)

-Lines 109-110: Why are some strains grown in large fermenters while others are grown in small batch cultures?

-Lines 164-168: need to provide more details on cell counting methods. Also, have the MPN and plating assay counts been cross-validated? Can the resulting data be integrated into a single dataset?

-For the radiation exposure, were cell numbers/biomass held equal between all cultures?

-Lines 181-184: Were your ICP-MS and ICP-OES data cross-validated to ensure that their resulting datasets can be combined?

-In Table 2, it is unclear to me why literature values are sometimes used for the D10 value but then other times the authors used their own measured values. Best practice would be to determine D10 values for all organisms under identical conditions using the same experimental methods. My understanding was that a primary objective of this work was to generate a representative dataset of microorganisms for which both D10 values and Mn/Fe ratios were measured using standardized approaches, thereby improving cross-taxon comparability relative to relying on literature values measured by various approaches. If the study is instead going to rely heavily on published D10 values, it might be more informative to integrate the newly generated data with the existing literature dataset (Tables 2 and 3) and present them together (for example, by combining Figures 2A and 2B) to leverage a larger and more comprehensive dataset.

-Fig 2B: could you color-code these plots by optimal growth temp as you did in Fig 2A? Also, do the 2B data originate from the values reported in Table 3?

-The discussion section doesn’t spend much time actually discussing the results of the study. Instead, a lot of text is devoted to reviewing the literature on radiation tolerance.. While this can be useful contextual information, the focus of the discussion should be on explaining your results and placing them in the broader literature. For example, why do your data in Fig 2A show no correlation between D10 and Mn/Fe but the literature data in Fig. 2B do show a correlation? What could be the cause of this discrepency? I will note that Lines 332-350 do a good job of critically examining the finding.

Minor Comments

-Lines 67-81: unclear if this section is talking about cellular exposure to X-rays or ionizing radiation in general.

-Lines 83-85: I don’t think this sentence makes sense with the ending of “e.g., enzymatic antioxidants” since there are other strategies discussed in the following sentences.

-Lines 95-96: need to define “oxidative stress reactions.”

-Table 1 refers to pH as one of the “optimal culture conditions,” but these pH values are not listed in the table

-In the Table 1 footnotes, some acronyms are defined but not others (MGG, SME, MAI, M, TGY, VM1)

-Line 144: What is “adapted PBS”?

-Figure 1A caption: both sets of bars are shaded…the literature data bars are striped

-Lines 236-237: I would not say that the D. radiodurans Mn/Fe ratio is two-fold higher than the literature value since you are comparing 0.25 to 0.32 +/- 0.13. The E. coli difference is more convincing, but you are still dealing with large error bars.

-Lines 292-344: This paragraph is overly long and easy to get lost in. Split into 2-3 different paragraphs

**Reviewer 2: Virginia Helena Albarracin, National University of Tucuman**

**Recommendation: Minor Revision**

his manuscript addresses an important and timely question in microbial radiobiology: whether the intracellular Mn/Fe ratio constitutes a universal predictor of ionising radiation tolerance across the tree of life. The authors showed experimental data from 21 organisms spanning Bacteria, Archaea, and Eukarya and compared them with an extensive compilation of literature values. Quantification of Mn and Fe using ICP-MS/OES, normalisation to biomass/protein/cell number, and replication are appropriately described. This generated a systematic and critical evaluation of a widely cited hypothesis.

In the well-written discussion, the authors integrate metal homeostasis, ROS detoxification, oxygen availability, temperature, and DNA repair into a coherent conceptual framework. The main conclusion — that no consistent correlation exists across domains — is scientifically sound, well supported by the presented data, and conceptually valuable. The work challenges an oversimplified paradigm derived largely from Deinococcus models and contributes to a more integrative, multifactorial understanding of radiation resistance.

In general, the manuscript is clearly written, technically sound, and relevant to the journal's readership. I recommend publication after moderate revisions.

Comments and recommendations

1. The Introduction is scientifically accurate and well-referenced, but it is longer than necessary for the core message of the paper. I suggest shortening it and focusing more directly on radiation-induced oxidative stress, the Mn/Fe hypothesis, previous evidence supporting it, and the specific knowledge gap this study addresses.

2. Currently, Table 2 (experimental data) and Table 3 (literature data) are both extensive and partially redundant in structure. I recommend combining Tables 2 and 3 and moving this combined table to the Supplementary Material.

3. Only two eukaryotic species (both Rhodotorula) are included. The manuscript should explicitly acknowledge that conclusions regarding Eukarya are preliminary and not broadly generalizable.

4. It would be useful to emphasise more clearly that Mn/Fe ratios represent a physiological snapshot under specific growth conditions rather than a fixed species trait.

5. Because organisms were irradiated under different redox conditions (oxic vs. anoxic), D10 values may not be strictly comparable across taxa. This limitation is mentioned but could be highlighted more explicitly as a potential confounding factor.

6. The study quantifies total intracellular Mn and Fe. A short statement noting that protective effects are likely mediated by specific pools (e.g., free Mn complexes vs. enzyme-bound metal) would further contextualise the findings.

7. Correct minor typographical inconsistencies (e.g., “Mn/Fe-radio” → “Mn/Fe-ratio” in the Introduction) and ensure consistent formatting of organism names and strain identifiers across tables.

**Associate Editor Recommendation: Major Revision**

Dear Dr. Beblo-Vranesevic,

Thank you for your submission to this special collection of FEMS Microbes. Both reviewers recognise the significance and timeliness of your investigation into the relationship between cellular metal homeostasis and radiation tolerance, but highlight the need for greater methodological transparency and structural focus.

They specifically highlight justifying your organism selection and the validity of integrating literature values with your new experimental data. Additionally, please streamline the Introduction and ensure the Discussion prioritises interpreting your specific results.

Detailed reviewer comments are provided below:

Reviewer 1

Beblo-Vranesevic and colleagues address the interesting question about the relationship between radiation tolerance and cellular metal homeostasis across microorganisms from all three domains of life. However, substantial clarification and additional methodological details are needed to improve the transparency and reproducibility of the findings. In particular, the rationale for organism selection, as well as significant differences in culturing approach, counting, and elemental analysis methods across the different strains, is needed. Additionally, further justification is required for integrating the newly generated Mn/Fe data with existing literature D10 values (rather than re-doing those measurements to ensure consistency across the entire dataset). The discussion section would also benefit from a stronger focus on interpreting the study’s own results (especially the discrepancies between the findings here and in the literature) rather than merely reviewing prior work. My detailed comments follow.

Major Comments

-It would be helpful to provide the rationale behind the selection of the organisms listed in Table 1. It would also be helpful to include additional metadata in this table: environment of origin of each strain, phylum (I think the authors may be going for phylogenetic breadth here), whether these are strict aerobes/facultative anaerobes/strict anaerobes (beyond just listing “aerobic” and “anaerobic”)

-Lines 109-110: Why are some strains grown in large fermenters while others are grown in small batch cultures?

-Lines 164-168: need to provide more details on cell counting methods. Also, have the MPN and plating assay counts been cross-validated? Can the resulting data be integrated into a single dataset?

-For the radiation exposure, were cell numbers/biomass held equal between all cultures?

-Lines 181-184: Were your ICP-MS and ICP-OES data cross-validated to ensure that their resulting datasets can be combined?

-In Table 2, it is unclear to me why literature values are sometimes used for the D10 value but then other times the authors used their own measured values. Best practice would be to determine D10 values for all organisms under identical conditions using the same experimental methods. My understanding was that a primary objective of this work was to generate a representative dataset of microorganisms for which both D10 values and Mn/Fe ratios were measured using standardized approaches, thereby improving cross-taxon comparability relative to relying on literature values measured by various approaches. If the study is instead going to rely heavily on published D10 values, it might be more informative to integrate the newly generated data with the existing literature dataset (Tables 2 and 3) and present them together (for example, by combining Figures 2A and 2B) to leverage a larger and more comprehensive dataset.

-Fig 2B: could you color-code these plots by optimal growth temp as you did in Fig 2A? Also, do the 2B data originate from the values reported in Table 3?

-The discussion section doesn’t spend much time actually discussing the results of the study. Instead, a lot of text is devoted to reviewing the literature on radiation tolerance.. While this can be useful contextual information, the focus of the discussion should be on explaining your results and placing them in the broader literature. For example, why do your data in Fig 2A show no correlation between D10 and Mn/Fe but the literature data in Fig. 2B do show a correlation? What could be the cause of this discrepency? I will note that Lines 332-350 do a good job of critically examining the finding.

Minor Comments

-Lines 67-81: unclear if this section is talking about cellular exposure to X-rays or ionizing radiation in general.

-Lines 83-85: I don’t think this sentence makes sense with the ending of “e.g., enzymatic antioxidants” since there are other strategies discussed in the following sentences.

-Lines 95-96: need to define “oxidative stress reactions.”

-Table 1 refers to pH as one of the “optimal culture conditions,” but these pH values are not listed in the table

-In the Table 1 footnotes, some acronyms are defined but not others (MGG, SME, MAI, M, TGY, VM1)

-Line 144: What is “adapted PBS”?

-Figure 1A caption: both sets of bars are shaded…the literature data bars are striped

-Lines 236-237: I would not say that the D. radiodurans Mn/Fe ratio is two-fold higher than the literature value since you are comparing 0.25 to 0.32 +/- 0.13. The E. coli difference is more convincing, but you are still dealing with large error bars.

-Lines 292-344: This paragraph is overly long and easy to get lost in. Split into 2-3 different paragraphs

Reviewer 2

This manuscript addresses an important and timely question in microbial radiobiology: whether the intracellular Mn/Fe ratio constitutes a universal predictor of ionising radiation tolerance across the tree of life. The authors showed experimental data from 21 organisms spanning Bacteria, Archaea, and Eukarya and compared them with an extensive compilation of literature values. Quantification of Mn and Fe using ICP-MS/OES, normalisation to biomass/protein/cell number, and replication are appropriately described. This generated a systematic and critical evaluation of a widely cited hypothesis.

In the well-written discussion, the authors integrate metal homeostasis, ROS detoxification, oxygen availability, temperature, and DNA repair into a coherent conceptual framework. The main conclusion — that no consistent correlation exists across domains — is scientifically sound, well supported by the presented data, and conceptually valuable. The work challenges an oversimplified paradigm derived largely from Deinococcus models and contributes to a more integrative, multifactorial understanding of radiation resistance.

In general, the manuscript is clearly written, technically sound, and relevant to the journal's readership. I recommend publication after moderate revisions.

Comments and recommendations

1. The Introduction is scientifically accurate and well-referenced, but it is longer than necessary for the core message of the paper. I suggest shortening it and focusing more directly on radiation-induced oxidative stress, the Mn/Fe hypothesis, previous evidence supporting it, and the specific knowledge gap this study addresses.

2. Currently, Table 2 (experimental data) and Table 3 (literature data) are both extensive and partially redundant in structure. I recommend combining Tables 2 and 3 and moving this combined table to the Supplementary Material.

3. Only two eukaryotic species (both Rhodotorula) are included. The manuscript should explicitly acknowledge that conclusions regarding Eukarya are preliminary and not broadly generalizable.

4. It would be useful to emphasise more clearly that Mn/Fe ratios represent a physiological snapshot under specific growth conditions rather than a fixed species trait.

5. Because organisms were irradiated under different redox conditions (oxic vs. anoxic), D10 values may not be strictly comparable across taxa. This limitation is mentioned but could be highlighted more explicitly as a potential confounding factor.

6. The study quantifies total intracellular Mn and Fe. A short statement noting that protective effects are likely mediated by specific pools (e.g., free Mn complexes vs. enzyme-bound metal) would further contextualise the findings.

7. Correct minor typographical inconsistencies (e.g., “Mn/Fe-radio” → “Mn/Fe-ratio” in the Introduction) and ensure consistent formatting of organism names and strain identifiers across tables.

**EIC Decision: Major Revision**

**Author's Response to Decision Letter for (FEMSMC-2025-072)**

Dear editor,

We have read with great interest the feedback of the reviewers and we believe they were very relevant in order to improve the manuscript.

We have considered all of the comments from the reviewers, which were very useful.

We will answer in detail to every suggestion in the following.

Both reviewers recognise the significance and timeliness of your investigation into the relationship between cellular metal homeostasis and radiation tolerance, but highlight the need for greater methodological transparency and structural focus.

They specifically highlight justifying your organism selection and the validity of integrating literature values with your new experimental data. Additionally, please streamline the Introduction and ensure the Discussion prioritises interpreting your specific results.

- These suggestions have been added to the manuscript, see also detailed answer to the reviewers 1 and 2.

Specific answer to reviewer 1:

Beblo-Vranesevic and colleagues address the interesting question about the relationship between radiation tolerance and cellular metal homeostasis across microorganisms from all three domains of life. However, substantial clarification and additional methodological details are needed to improve the transparency and reproducibility of the findings. In particular, the rationale for organism selection, as well as significant differences in culturing approach, counting, and elemental analysis methods across the different strains, is needed. Additionally, further justification is required for integrating the newly generated Mn/Fe data with existing literature D10 values (rather than re-doing those measurements to ensure consistency across the entire dataset). The discussion section would also benefit from a stronger focus on interpreting the study’s own results (especially the discrepancies between the findings here and in the literature) rather than merely reviewing prior work. My detailed comments follow.

- Organisms were chosen based on previous desiccation/radiation results, suggesting a possible correlation. However, our investigation did not uncover any correlation (Beblo-Vranesevic, K., et al. (2018). Lack of correlation between desiccation and radiation tolerance in microorganisms from diverse extreme environments tested under anoxic conditions. FEMS microbiology letters, 365(6), fny044). Subsequently, we examined the Mn/Fe-ratio of these strains to determine if a correlation was present. It appears that, at least for some of the strains we studied, no correlation exists.

- Culture approach: For the measurement, a substantial amount of cells is required. Some of the strains only grow to low density, so high-volume fermenters were utilized to increase cell mass.

- Counting: Certain organisms cannot grow on solid surfaces (agar plates), so survival determination was investigated using dissolution techniques. Both techniques were compared and cross-validated. The results from both methods are comparable.

- D10-values and Mn/Fe-ratios were determined in cases where literature data was missing, filling in the gaps. To cross-validate our techniques, we compared our results with existing data and strains. We observed differences to some extent, but we deemed them as not significant. If differences were present, we attempted to explain them. For instance, varying levels of Mn/Fe may be attributed to differences in cultivation conditions (such as media).

- We streamlined the discussion section, condensing it without sacrificing scientific depth.

Major Comments

-It would be helpful to provide the rationale behind the selection of the organisms listed in Table 1. It would also be helpful to include additional metadata in this table: environment of origin of each strain, phylum (I think the authors may be going for phylogenetic breadth here), whether these are strict aerobes/facultative anaerobes/strict anaerobes (beyond just listing “aerobic” and “anaerobic”)

- Organisms were chosen based on previous desiccation/radiation results, suggesting a possible correlation. However, our investigation did not uncover any correlation (Beblo-Vranesevic, K., et al. (2018). Lack of correlation between desiccation and radiation tolerance in microorganisms from diverse extreme environments tested under anoxic conditions. FEMS microbiology letters, 365(6), fny044). Subsequently, we examined the Mn/Fe-ratio of these strains to determine if a correlation was present. It appears that, at least for some of the strains we studied, no correlation exists.

- Metadata, such as detailed O2 metabolism, strain origin (environment), and phylum, were included in Table 1.

-Lines 109-110: Why are some strains grown in large fermenters while others are grown in small batch cultures?

- Culture approach: For the measurement, a substantial amount of cells is required. Some of the strains only grow to low density, so high-volume fermenters were utilized to increase cell mass. The rationale behind this is now included in the manuscript (L114-117).

-Lines 164-168: need to provide more details on cell counting methods. Also, have the MPN and plating assay counts been cross-validated? Can the resulting data be integrated into a single dataset?

- Counting. Some of the organisms cannot grow on solid surfaces (agar plates) and the determination of survival was investigated by dilution techniques. Comparison of both techniques were applied before and cross validated. The results of both techniques are comparable. Details have been included in the manuscript (L175-177)

-For the radiation exposure, were cell numbers/biomass held equal between all cultures?

- Mostly, we irradiated 107-108 cells per mL, and the treatment was consistent for all tested strains (L162). Each radiation exposure was conducted as a separate experiment, with an internal (dark) control, yielding a survival curve. The D10-value was then determined and compared to literature data. Additional details have been provided in the manuscript (L180-183).

-Lines 181-184: Were your ICP-MS and ICP-OES data cross-validated to ensure that their resulting datasets can be combined?

- Both methods focus on measuring ion concentrations. We primarily utilized ICP-OES, which enables a higher number of samples to be processed. Furthermore, in samples where we measured levels previously analyzed with ICP-MS, we obtained similar results (e.g., P. furiosus). Consequently, we concluded that both methods are comparable. Information regarding their comparability has been included in the manuscript (L236-241). Additionally, we compared the concentration within a single species relative to cell mass. The resulting ratio should be consistent with both methods.

-In Table 2, it is unclear to me why literature values are sometimes used for the D10 value but then other times the authors used their own measured values. Best practice would be to determine D10 values for all organisms under identical conditions using the same experimental methods.

- We determined the D10-values where no literature data was available, thereby filling the gaps. For the strains mentioned by Beblo et al. 2011, Bauermeister et al. 2011, and Siems et al. 2022 and those investigated in this study, the conditions were identical as the D10-values were all determined in our labs.

-My understanding was that a primary objective of this work was to generate a representative dataset of microorganisms for which both D10 values and Mn/Fe ratios were measured using standardized approaches, thereby improving cross-taxon comparability relative to relying on literature values measured by various approaches. If the study is instead going to rely heavily on published D10 values, it might be more informative to integrate the newly generated data with the existing literature dataset (Tables 2 and 3) and present them together (for example, by combining Figures 2A and 2B) to leverage a larger and more comprehensive dataset.

- Table 2 and Table 3 have now been merged. The new datasets (filling the gaps) are highlighted in bold.

- In Fig. 2, we believe separate panels would be better. Panel A mainly displays data from our investigation of Mn/Fe in this study, while Panel B shows only literature data where Mn/Fe-values already existed. The figures are of the same size and dimensions, making them directly comparable and less crowded than if they were combined into one panel.

-Fig 2B: could you color-code these plots by optimal growth temp as you did in Fig 2A? Also, do the 2B data originate from the values reported in Table 3?

- Fig 2B now has the same color code as Fig. 2A. Thank you for this suggestion, which enhances the comparability of the two figures.

-The discussion section doesn’t spend much time actually discussing the results of the study. Instead, a lot of text is devoted to reviewing the literature on radiation tolerance. While this can be useful contextual information, the focus of the discussion should be on explaining your results and placing them in the broader literature. For example, why do your data in Fig 2A show no correlation between D10 and Mn/Fe but the literature data in Fig. 2B do show a correlation? What could be the cause of this discrepency? I will note that Lines 332-350 do a good job of critically examining the finding.

- We aimed to emphasize our findings on Mn/Fe-ratios. However, we cannot definitively explain the discrepancy in the various results. While there may be a correlation between the Mn/Fe ratio and tolerance to ionizing radiation in some strains, as indicated by decades of research, this relationship does not hold true for all (newly tested) strains.

Minor Comments

-Lines 67-81: unclear if this section is talking about cellular exposure to X-rays or ionizing radiation in general.

- Rephrased and discussing ionizing radiation in general.

-Lines 83-85: I don’t think this sentence makes sense with the ending of “e.g., enzymatic antioxidants” since there are other strategies discussed in the following sentences.

- Rephrased: Initially described enzymatic antioxidants, followed by the description of non-enzymatic antioxidants (e.g., pigments). This was later incorporated into the discussion section (L. 348-351).

-Lines 95-96: need to define “oxidative stress reactions.”

- Rephrased.

-Table 1 refers to pH as one of the “optimal culture conditions,” but these pH values are not listed in the table

-In the Table 1 footnotes, some acronyms are defined but not others (MGG, SME, MAI, M, TGY, VM1)

- Table 1 and its heading and footnotes have been restructured.

- Metadata such as detailed O2 metabolism, strain origin (environment), and phylum were included in Table 1. We have removed the abbreviations of the media as they are not relevant in the context of the study.

-Line 144: What is “adapted PBS”?

- PBS was adjusted in terms of salinity for each strain. The term is explained the first time it is mentioned at L138.

-Figure 1A caption: both sets of bars are shaded…the literature data bars are striped

- Changed into striped

-Lines 236-237: I would not say that the D. radiodurans Mn/Fe ratio is two-fold higher than the literature value since you are comparing 0.25 to 0.32 +/- 0.13. The E. coli difference is more convincing, but you are still dealing with large error bars.

- We reviewed and recalculated the data for D. radiodurans. There was an error in the calculation of the errors previously. Apologies! The paragraph has been rephrased to now state that D. radiodurans and E. coli have higher values, whereas the amount in P. furiosus falls within the same range (L.236-241).

-Lines 292-344: This paragraph is overly long and easy to get lost in. Split into 2-3 different paragraphs

- - Paragraph has now several sections.

Reviewer 2

This manuscript addresses an important and timely question in microbial radiobiology: whether the intracellular Mn/Fe ratio constitutes a universal predictor of ionising radiation tolerance across the tree of life. The authors showed experimental data from 21 organisms spanning Bacteria, Archaea, and Eukarya and compared them with an extensive compilation of literature values. Quantification of Mn and Fe using ICP-MS/OES, normalisation to biomass/protein/cell number, and replication are appropriately described. This generated a systematic and critical evaluation of a widely cited hypothesis.

In the well-written discussion, the authors integrate metal homeostasis, ROS detoxification, oxygen availability, temperature, and DNA repair into a coherent conceptual framework. The main conclusion — that no consistent correlation exists across domains — is scientifically sound, well supported by the presented data, and conceptually valuable. The work challenges an oversimplified paradigm derived largely from Deinococcus models and contributes to a more integrative, multifactorial understanding of radiation resistance.

In general, the manuscript is clearly written, technically sound, and relevant to the journal's readership. I recommend publication after moderate revisions.

Comments and recommendations

1. The Introduction is scientifically accurate and well-referenced, but it is longer than necessary for the core message of the paper. I suggest shortening it and focusing more directly on radiation-induced oxidative stress, the Mn/Fe hypothesis, previous evidence supporting it, and the specific knowledge gap this study addresses.

- We condensed the introduction without losing scientific depth.

2. Currently, Table 2 (experimental data) and Table 3 (literature data) are both extensive and partially redundant in structure. I recommend combining Tables 2 and 3 and moving this combined table to the Supplementary Material.

- Table 2 and Table 3 have been merged. We believe it is beneficial to keep the table in the main manuscript as it serves as a useful tool to pinpoint specific data in Fig. 2 for individual organisms without having the need to access the supplementary files which could be overlooked by the readers.

3. Only two eukaryotic species (both Rhodotorula) are included. The manuscript should explicitly acknowledge that conclusions regarding Eukarya are preliminary and not broadly generalizable.

- The sections discussing Eukaryotes are now more specific, focusing primarily on yeasts with two examples investigated.

4. It would be useful to emphasise more clearly that Mn/Fe ratios represent a physiological snapshot under specific growth conditions rather than a fixed species trait.

- A section addressing growth conditions and discussing the snapshot is now included in the discussion section (L. 300-308).

5. Because organisms were irradiated under different redox conditions (oxic vs. anoxic), D10 values may not be strictly comparable across taxa. This limitation is mentioned but could be highlighted more explicitly as a potential confounding factor.

- A statement about the redox conditions has been added. Now providing better context (L364).

6. The study quantifies total intracellular Mn and Fe. A short statement noting that protective effects are likely mediated by specific pools (e.g., free Mn complexes vs. enzyme-bound metal) would further contextualise the findings.

- A statement about Mn-free complexes has been added to the discussion section (L348).

7. Correct minor typographical inconsistencies (e.g., “Mn/Fe-radio” → “Mn/Fe-ratio” in the Introduction) and ensure consistent formatting of organism names and strain identifiers across tables.

- Typos have been corrected, and the organisms should now be consistently shown in the same format throughout the manuscript.

We would like to thank once again the reviewers for their critical comments which have improved the manuscript. All changes in the revised manuscript and in the response to the reviewers are highlighted in yellow background.

Looking forward to your final decision.

Best regards,

Kristina Beblo-Vranesevic

**Reviews of manuscript FEMSMC-2025-072.R1**

**Reviewer 1**

**Recommendation: Minor Revision**

The authors have made significant revisions and clarifications since the original version of the manuscript, which I greatly appreciate. I found them to be very responsive to the reviewer comments. Overall, though, I continue to have doubts about whether the data collected here under really varied growth conditions can genuinely be integrated with the literature data (also collected under varied growth conditions). Figure 1 seems to suggest “no” or “it may work better for some organisms than others”. With that said, I also don’t know that there is really anything that the authors could be doing better since I think this is just an inherent limitation of the study. I think what is needed is an extensive discussion of caveats and limitations within the discussion section. This was discussed to some extent in Lines 295-303 but I’d like to see an explicit “limitations” section that addresses the various limitations of the study (different growth conditions, varied redox conditions, different ways of measuring biomass, limited number of eukaryotes) and perhaps a mention of future directions for research that could help overcome these limitations. Maybe a separate “Limitations and Future Directions” section would make this more explicit?

Some additional comments:

Line 94 – the Mn/Fe ratio is modified in response to ROS, but typically Mn increases and Fe decreases. This should be explicitly stated. Right now, your text says that both increase, which would not actually impact the ratio.

Line 100 – need a space in “modelorganisms”

Line 100-101 – This sentence is awkward. I can tell you are transitioning to talking about your experimental choices (i.e., which organisms to work with), but it’s kind of randomly placed here and without context. It might work better in the next paragraph where you start talking about your study.

Table 1 – Can you list the pH used for your growth experiments?

Line 173 – I would like to see this cross validation provided in the supplemental information. This is critical to the validity of your results. Hopefully it was for more than just one organism.

Figure 1 – there needs to be some explanation as to why your measurements for D. radiodurans and E. coli differ a lot from the literature values. To me, this suggests that a lot of variability is introduced by culturing and so the datasets cannot really be integrated.

Line 271 – I don’t really know that you have a large enough of a sample size to really be able to determine if there is any relationship to temperature..maybe for the mesophiles and hyperthermophiles.

Line 295 – 321 – This paragraph is overly long

**Reviewer 2: Virginia Helena Albarracin, National University of Tucuman**

**Recommendation: Accept**

The authors have clearly improved the quality of the manuscript. Likewise, they have responded and addressed all my comments.

**Associate Editor Recommendation: Minor Revision**

Thank you for providing your revised manuscript, complete with edits and responses to the reviewers comments. Both reviewers agree that the manuscript is signficiantly improved - one suggestion to fully contextualise the study would be addition of a Limitations and Future Directions section to the discussion, building on your existing discussion to address possible limitations of the study and to address future experimentation that could further advance the field.

**EIC Decision: Minor Revision**

**Author's Response to Decision Letter for (FEMSMC-2025-072.R1)**

Dear editors,

- We thank the reviewers for their positive and helpful feedback. In the following, we address each comment step by step. All new changes are marked in green within the manuscript. The changes from the first round of revision are still marked in yellow.

Reviewers' Comments to Author/s:

Reviewer: 1

Comments to the Author

The authors have made significant revisions and clarifications since the original version of the manuscript, which I greatly appreciate. I found them to be very responsive to the reviewer comments. Overall, though, I continue to have doubts about whether the data collected here under really varied growth conditions can genuinely be integrated with the literature data (also collected under varied growth conditions). Figure 1 seems to suggest “no” or “it may work better for some organisms than others”. With that said, I also don’t know that there is really anything that the authors could be doing better since I think this is just an inherent limitation of the study. I think what is needed is an extensive discussion of caveats and limitations within the discussion section. This was discussed to some extent in Lines 295-303 but I’d like to see an explicit “limitations” section that addresses the various limitations of the study (different growth conditions, varied redox conditions, different ways of measuring biomass, limited number of eukaryotes) and perhaps a mention of future directions for research that could help overcome these limitations. Maybe a separate “Limitations and Future Directions” section would make this more explicit?

- We thank the reviewer for the positive and helpful feedback, as well as for the constructive suggestion to further elaborate on the limitations of the study. We fully agree that these aspects are important for the interpretation of our results. A separate section has been included. You can find it now at the very end of the manuscript within the conclusion section. This part includes discussion of methodological differences, strain-dependent effects, differences in the measurement of survivability, and variations in growth conditions (including redox conditions and temperature). It also addresses the limited number of samples, particularly with respect to eukaryotic cells.

Some additional comments:

Line 94 – the Mn/Fe ratio is modified in response to ROS, but typically Mn increases and Fe decreases. This should be explicitly stated. Right now, your text says that both increase, which would not actually impact the ratio.

- This sentence has been revised and changed.

Line 100 – need a space in “modelorganisms”

- Space has been added.

Line 100-101 – This sentence is awkward. I can tell you are transitioning to talking about your experimental choices (i.e., which organisms to work with), but it’s kind of randomly placed here and without context. It might work better in the next paragraph where you start talking about your study.

- Thank you for the suggestion. Sentence has been shifted to the next paragraph.

Table 1 – Can you list the pH used for your growth experiments?

- pH is now included in the table.

Line 173 – I would like to see this cross validation provided in the supplemental information. This is critical to the validity of your results. Hopefully it was for more than just one organism.

- Since these evaluations were not originally performed within the scope of the present study, we would prefer not to include them in the supplementary material.

However, we would like to clarify that the approach used here is based on established and widely accepted methods. MPN-assays are used for strains not able to grow on plates and plating methods are applied for strains capable of growth on solid media. This represents standard practice in microbiology for estimating viable cell numbers depending on the growth characteristics of the organism.

Importantly, each experiment also included an internal non-treated control, allowing for normalization and comparison against the initial cell input independently of the treatment conditions. This provides an additional layer of validation for the quantitative results.

- In addition, we have now added several relevant references from previous works where both plating and MPN-based techniques have been successfully applied, particularly for Buttiauxella and, to some extent, Salinisphaera, among other organisms.

Figure 1 – there needs to be some explanation as to why your measurements for D. radiodurans and E. coli differ a lot from the literature values. To me, this suggests that a lot of variability is introduced by culturing and so the datasets cannot really be integrated.

- We agree that these deviations warrant clarification. Several factors may contribute to the observed differences. First, cultivation conditions can significantly influence the measured outcomes, even under seemingly comparable experimental setups. Second, methodological differences are relevant (ICP-MS vs. ICP-OES). Third, and potentially most importantly, strain-specific variability must be considered. Even when using the same strain designation (D. radiodurans R1), lab-specific differences can arise over time due to strain handling and maintenance. This is further supported by the observed variability among different Deinococcus strains in Figure 1B, which show slightly different behaviors. Additionally, it should be noted that Figure 1 presents a linear representation of the data, whereas many literature sources use logarithmic scaling, which can visually reduce apparent differences.

- We have now added one sentence in the manuscript explicitly mentioning these possible reasons to clarify the interpretation of the observed deviations.

Line 271 – I don’t really know that you have a large enough of a sample size to really be able to determine if there is any relationship to temperature..maybe for the mesophiles and hyperthermophiles.

- We fully agree that the current sample size is too limited to draw statistically robust conclusions regarding a relationship between temperature and the measured parameters. Our intention in this section was to explore whether any obvious trends related to different growth conditions (incl. temperature or pH) could be observed in the available dataset. However, as correctly pointed out, no consistent dependence was found, which is also stated in the manuscript.

Line 295 – 321 – This paragraph is overly long

- Paragraph has been split into shorter sections.

Reviewer: 2

Comments to the Author

The authors have clearly improved the quality of the manuscript. Likewise, they have responded and addressed all my comments.

We would like to thank once again the reviewers for their critical comments which have improved the manuscript. All changes in the revised manuscript and in the response to the reviewers are highlighted in green background.

Looking forward to your final decision.

Best regards,

Kristina Beblo-Vranesevic

**Reviews of manuscript FEMSMC-2025-072.R2**

**Associate Editor Recommendation: Accept**

Many thanks to the authors for their detailed revisions to the manuscript and addition of a limitations and future research section.

**EIC Decision: Accept**
